# Cardiovascular and Neurological Diseases and Association with Helicobacter Pylori Infection—An Overview

**DOI:** 10.3390/diagnostics14161781

**Published:** 2024-08-15

**Authors:** Vlad Pădureanu, Dalia Dop, Daniel Cosmin Caragea, Dumitru Rădulescu, Rodica Pădureanu, Mircea-Cătălin Forțofoiu

**Affiliations:** 1 Department of Internal Medicine, University of Medicine and Pharmacy Craiova, 200349 Craiova, Romania; vlad.padureanu@umfcv.ro (V.P.); catalin.fortofoiu@umfcv.ro (M.-C.F.); 2 Department of Pediatrics, University of Medicine and Pharmacy Craiova, 200349 Craiova, Romania; dalia.dop@umfcv.ro; 3 Department of Nephrology, University of Medicine and Pharmacy Craiova, 200349 Craiova, Romania; daniel.caragea@umfcv.ro; 4 Department of Surgery, University of Medicine and Pharmacy Craiova, 200349 Craiova, Romania

**Keywords:** *helicobacter pylori*, stroke, myocardial infarction

## Abstract

This article investigates the link between *Helicobacter pylori* (*H. pylori*) infection and cardiovascular and neurological disorders. Recent research suggests that *H. pylori* may play a role in cardiovascular diseases like atherosclerosis, myocardial infarction, and stroke, as well as neurological diseases including Alzheimer’s disease, multiple sclerosis, and Parkinson’s disease. Cardiovascular Diseases: *H. pylori* induces endothelial dysfunction and chronic inflammation, promoting atherosclerotic plaque formation and other cardiac complications. High infection prevalence in cardiovascular patients implies that systemic inflammation from *H. pylori* accelerates disease progression. Eradication therapies combined with anti-inflammatory and lipid-lowering treatments may reduce cardiovascular risk. Neurological Diseases: *H. pylori* may contribute to Alzheimer’s, multiple sclerosis, and Parkinson’s through systemic inflammation, neuroinflammation, and autoimmune responses. Increased infection prevalence in these patients suggests bacterial involvement in disease pathogenesis. The eradication of *H. pylori* could reduce neuroinflammation and improve outcomes. Discussions and Future Research: Managing *H. pylori* infection in clinical practice could impact public health and treatment approaches. Further research is needed to clarify these relationships. Longitudinal and mechanistic studies are essential to fully understand *H. pylori*’s role in these conditions. Conclusions: *H. pylori* infection is a potential risk factor for various cardiovascular and neurological conditions. Additional research is critical for developing effective prevention and treatment strategies. Targeted therapies, including *H. pylori* eradication combined with anti-inflammatory treatments, could improve clinical outcomes. These findings highlight the need for an integrated clinical approach to include *H. pylori* evaluation and treatment.

## 1. Introduction

*Helicobacter pylori* (*H. pylori*) is a Gram-negative, spiral-shaped, flagellated, microaerophilic bacterium primarily colonizing the human stomach. Approximately half of the global population harbors this infection [1]. The prevalence of *H. pylori* varies significantly by geographic location, age, ethnicity, and socioeconomic status, with higher rates in developing countries and among lower socioeconomic groups [2,3,4]. The bacterium’s unique features enable it to thrive in the stomach’s acidic environment. Transmission primarily occurs through the oro-fecal route via contaminated food and water [5], and potentially through oro-oral routes, as evidenced by its presence in dental plaque and saliva [6].

Emerging research has established links between *H. pylori* infection and various extragastric conditions, notably cardiovascular and neurological disorders, including increased risks of ischemic stroke and atherosclerosis due to systemic inflammation and metabolic disturbances [7,8,9,10]. The exact mechanisms remain unclear but are thought to involve a combination of host genetic factors (e.g., TNFα gene polymorphism, IL1B gene cluster), bacterial virulence factors (e.g., CagA, urease, VacA, babA2), and environmental influences (e.g., socioeconomic status, nutrition, toxin exposure) [11].

Numerous studies have reported a higher incidence of cardiovascular diseases (CVD) among those infected with *H. pylori*. For instance, research in Korea revealed that *H. pylori*-seropositive individuals exhibit higher levels of total cholesterol, triglycerides, and LDL cholesterol and lower HDL cholesterol compared to seronegative individuals [12]. Conversely, a study in Germany found no increased risk of myocardial infarction or overall mortality associated with *H. pylori* infection, though CagA-positive strains were inversely related to cardiovascular mortality [13]. In Northern Ireland, *H. pylori* infection was prevalent in 50.5% of the population and associated with cardiovascular risk factors such as age and smoking [14]. Additionally, a study in Afghanistan linked *H. pylori* infection with higher rates of diabetes mellitus and elevated BMI [15].

Chronic inflammation induced by *H. pylori* can lead to endothelial dysfunction, a precursor to atherosclerosis. Evidence supports systemic inflammation as a key mechanism connecting *H. pylori* to cardiovascular diseases. For example, one study associated *H. pylori* infection with elevated inflammatory markers like C-reactive protein (CRP) and interleukin-6 (IL-6), which contribute to endothelial dysfunction and atherosclerosis progression [16]. Another study highlighted persistent stomach inflammation due to *H. pylori*, with local inflammatory mediators such as IL-8, TNF-α, and IL-1β entering the systemic circulation and exacerbating systemic inflammation [17].

In the realm of neurological diseases, *H. pylori* infection might influence pathogenesis through systemic inflammation and autoimmune mechanisms. The immune response to *H. pylori* involves complex interactions between cytokines and immune cells, contributing to systemic inflammation and autoimmunity. Pro-inflammatory cytokines such as IL-6 and TNF-α, released during *H. pylori* infection, are implicated in these processes [18]. Furthermore, *H. pylori* can trigger autoantibody production that cross-reacts with host antigens, causing tissue damage and systemic inflammation, potentially leading to autoimmune and inflammatory diseases [19].

The connection between *H. pylori* infection and extragastric diseases was first documented by Mendall et al. in 1994 [20]. This relationship remains both intriguing and controversial, with various extragastric clinical symptoms reported in association with *H. pylori* infection. Understanding these associations’ clinical importance could significantly impact public health and therapeutic strategies. This article aims to synthesize the current literature on the association between *H. pylori* infection and cardiovascular and neurological disorders, elucidating the pathogenic mechanisms and clinical implications of this relationship.

## 2. Materials and Methods

A narrative review was conducted to synthesize the current literature on the associations between *H. pylori* infection and cardiovascular and neurological diseases. The PubMed and Scopus databases were searched using the terms: “*Helicobacter pylori* infection”, “cardiovascular diseases”, and “neurological diseases”. The search encompassed all English-language articles without date restrictions to ensure a comprehensive coverage of the relevant literature. The search was performed on 28 May 2024. Letters, observations, and viewpoints were excluded from the search.

Each identified article was individually evaluated by the authors for relevance, focusing on the methodological design, study population, diagnostic methods for *H. pylori* infection, and outcome measures. Only studies providing robust evidence and relevant statistical data were included in the analysis. The authenticity and methodological rigor of each study were critical criteria for selection to ensure the quality and relevance of the review.

To identify additional relevant articles, the references of initially selected studies were also examined. All studies were independently reviewed by two authors, and any discrepancies in relevance assessments were resolved through discussion and consensus.

This methodology facilitated a rigorous and systematic selection of studies, ensuring that the review provides a comprehensive and reliable presentation of the associations between Helicobacter pylori infection and cardiovascular and neurological diseases.

## 3. Cardiovascular Diseases (CVDs)

### 3.1. Introduction to CVDs Associated with H. Pylori

#### 3.1.1. Epidemiological Context

The association between *H. pylori* infection and CVDs has drawn significant interest. Understanding the precise role of *H. pylori* in the pathogenesis of CVDs, which encompasses myocardial infarction, stroke, and atherosclerotic coronary artery disease (CAD), has made considerable progress despite certain inherent challenges.

Mendall et al. were pioneers in this field by reporting a significant correlation between *H. pylori* infection and the development of CAD in men aged 45–65 years [20]. The prevalence of *H. pylori* infection varies significantly across geographic regions and subpopulations with CVDs, being higher in Africa (70.1%) and lower in Oceania (24.4%) [21]. In Europe, prevalence ranges from 3.4% in Iceland to 69.2% in Estonia [22]. In the USA, the overall prevalence of infection was 32.5%, with higher rates among non-Hispanic Blacks (52.7%) and Mexican Americans (61.6%) compared to non-Hispanic Whites (26.2%) [23].

Hamrah et al. demonstrated in Afghanistan that *H. pylori* infection was significantly associated with diabetes mellitus and an increased body mass index [15]. In Japan, Kinjo et al. found that the prevalence of *H. pylori* infection in patients with acute myocardial infarction was significantly higher in individuals < 55 years old compared to the control group (58.7% vs. 43.3%) [24]. In Korea, Sung et al. demonstrated that *H. pylori* infection was associated with increased levels of total cholesterol, triglycerides, and LDL cholesterol and decreased HDL cholesterol levels [12]. Wan et al. revealed a link between *H. pylori* infection and cardiovascular risk factors, including hypertension [25].

#### 3.1.2. Risk Factors and Pathogenic Mechanisms

*H. pylori* infection is associated with various cardiovascular conditions, with risk factors such as smoking, diet, genetics, and environmental influences playing a crucial role in this interaction. Kim et al. demonstrated that *H. pylori* infection was associated with higher levels of total cholesterol and LDL-C and lower levels of HDL-C [26]. Findings corroborated these results, showing that seropositivity for *H. pylori* was associated with elevated triglyceride and reduced HDL-C levels, independent of peptic ulcer presence [12].

Further research identified a correlation between *H. pylori* infection and elevated levels of inflammatory markers, such as C-reactive protein (CRP), in patients with type 2 diabetes [27]. Strachan et al. noted that *H. pylori* infection is associated with a lower socioeconomic status in childhood and adulthood [28]. In Central Africa, studies found that high levels of anti-*H. pylori* IgG antibodies were correlated with traditional cardiovascular risk factors, such as hypertension and elevated total cholesterol levels, particularly in men [29]. Additional research showed that genetic and environmental factors can modulate the pathogenic risk of *H. pylori* infection, influencing a predisposition to cardiovascular complications [30].

*H. pylori* infection interacts with various cardiovascular risk factors, including dyslipidemia, diabetes mellitus, systemic inflammation, socioeconomic status, and genetic and environmental influences, contributing to the development of CVDs. Understanding these interactions is essential for developing effective prevention and treatment strategies.

#### 3.1.3. Pathogenic Mechanisms of *H. pylori* Infection

*H. pylori* infection contributes to various cardiovascular complications through complex and multifactorial mechanisms. Studies have shown that *H. pylori* infection induced a chronic inflammatory response in the gastric mucosa, resulting in the release of pro-inflammatory cytokines, such as interleukin-8 [IL-8] and tumor necrosis factor-α (TNF-α), which, in turn, promoted systemic inflammation and contributed to atherosclerosis [31]. Camilo et al. demonstrated that *H. pylori* infection can lead to endothelial dysfunction via the generation of reactive oxygen species (ROS) and reducing nitric oxide (NO) bioavailability, which in turn causes vasoconstriction, thrombosis, and atherosclerosis [32].

It has been found that *H. pylori* induced platelet aggregation by binding to von Willebrand factor (vWF) and interacting with the GPIb receptor on platelets, potentially leading to thrombosis and cardiovascular complications [33]. Research also emphasized that *H. pylori* proteins such as CagA and VacA can trigger autoimmune reactions through molecular mimicry, activating T and B lymphocytes, and inducing the production of antibodies that may attack endothelial cells and other vascular structures [31]. Liu et al. showed that *H. pylori* modulated the expression of microRNAs that regulate inflammatory responses, notably increasing the expression of miR-146a, which negatively regulated inflammatory pathways by targeting IRAK1 and TRAF6 [34]. Additional findings demonstrated that *H. pylori* induced oxidative stress, causing DNA damage and activating DNA repair pathways, such as homologous recombination, contributing to genomic instability and the development of CVDs [35].

In summary, *H. pylori* infection contributes to CVDs through mechanisms involving chronic inflammation, endothelial dysfunction, interactions with coagulation factors, autoimmunity, microRNA modulation, and oxidative stress. Understanding these mechanisms can help develop more effective prevention and treatment strategies for CVDs associated with *H. pylori* infection.

#### 3.1.4. Longitudinal Studies and Temporal Evolution of CVDs

The temporal evolution of CVDs in the context of *H. pylori* infection has been examined through longitudinal studies tracking the progression of infected patients over time. One study reported, in a 13-year-long study of middle-aged men in the UK, that seropositivity for *H. pylori* was associated with an increased risk of myocardial infarction and stroke, although this association was significantly influenced by social class and other major cardiovascular risk factors [36].

Another cohort study of 9953 German adults followed for 5 years found that *H. pylori* infection, including CagA-positive strains, was not associated with an increased risk of myocardial infarction, stroke, or overall mortality. Interestingly, infection with CagA-positive strains was inversely associated with cardiovascular mortality [13]. Similarly, research in South Wales observed that *H. pylori* infection was associated with higher overall and cardiovascular mortality; however, these associations were not significant after adjusting for cardiovascular risk factors and socioeconomic status [28].

In diabetic patients, it was demonstrated that *H. pylori* infection was associated with a higher prevalence of coronary artery disease and cerebrovascular diseases compared to uninfected patients [37]. Lee et al. found in a healthy population that current *H. pylori* infection was associated with significant subclinical coronary stenosis, with the *H. pylori*-positive group showing a higher incidence of significant coronary stenosis after adjusting for confounding factors [38]. In contrast, Lin et al. reported no significant association between *H. pylori* infection and the risk of cardiovascular mortality or stroke in a Japanese cohort [39].

These longitudinal and comparative studies suggest a complex relationship between *H. pylori* infection and CVDs, with certain studies indicating a significant association while others do not find an association. Cardiovascular risk factors and socioeconomic status seem to influence these relationships, and the underlying mechanisms require further investigation.

### 3.2. Atherosclerosis and H. pylori: Pathophysiological Mechanisms

#### 3.2.1. Formation of Atherosclerotic Plaques

Atherosclerosis is characterized by the formation of unstable plaques within the vascular endothelium that are prone to rupture. These plaques develop when endothelial function is compromised, leading to vascular wall remodeling, increased blood pressure, localized inflammation, and blood coagulation. Plaque rupture can disrupt blood flow, resulting in myocardial infarction. The notion that infectious agents might contribute to the pathophysiology of atherosclerotic coronary artery disease (CAD) is relatively recent, arising from observations that certain individuals develop CAD without traditional risk factors.

#### 3.2.2. Endothelial Dysfunction and Chronic Inflammation

*H. pylori*, commonly associated with gastric infections, may also contribute to the development of atherosclerosis via mechanisms involving endothelial dysfunction and chronic inflammation. Research has demonstrated that chronic *H. pylori* infection is linked to endothelial dysfunction and systemic vascular inflammation [40]. Further studies further showed that eradicating *H. pylori* infection significantly improved endothelial function in infected patients [41].

Chronic *H. pylori* infections can lead to systemic inflammation, which contributes to the development of atherosclerosis. Adiloğlu et al. found that seropositivity for *H. pylori* is associated with elevated levels of CRP and IL-6 [42]. Additionally, *H. pylori* infection can affect endothelial function through exosomal mechanisms. Xia et al. showed that *H. pylori* inhibited endothelial function via the release of exosomes derived from gastric epithelial cells [43].

#### 3.2.3. Chronic Inflammation and the Role of Pathogen Recognition Receptors [PRRs]

Chronic inflammation induced by *H. pylori* plays a crucial role in the development and progression of atherosclerosis. Research revealed that chronic *H. pylori* infection was associated with elevated levels of hs-CRP (high-sensitivity C-reactive protein), an inflammatory marker involved in the pathogenesis of CAD [44]. Further studies showed that *H. pylori*-induced chronic inflammation is linked to changes in lipid profiles, thereby increasing the risk of atherosclerosis [45].

*H. pylori* infection is associated with increased levels of IL-6 and CRP, suggesting that chronic inflammation significantly contributes to the progression of atherosclerosis, as noted by Adiloğlu et al. [42]. Sawayama et al. further highlighted the impact of chronic inflammation on the cardiovascular system, showing an association between chronic *H. pylori* infection and an increased risk of ischemic stroke [46].

*H. pylori* antigens can bind to LDL/oxLDL cholesterol or directly interact with the vascular endothelium. By activating the immune system or epithelial cells through PRRs, which recognize pathogen-associated molecular patterns, subacute inflammation in chronic disorders like CAD can be identified. Tongtawee et al. emphasized this interaction [47]. In atherosclerotic lesions, both endothelial cells and macrophages upregulate PRRs to detect bacterial lipopolysaccharides (LPSs), such as TLR4, CD14, and TLR2, according to various findings [48]. Proposals suggest a potential pathophysiological link between lipids, *H. pylori* infection, inflammatory state, and CAD, based on the upregulation of TLR4 expression on the surface of macrophages caused by oxidized LDL [49].

#### 3.2.4. Inflammatory Markers and Molecular Mimicry

The association between CAD and significant inflammatory markers, such as TNF-α, IL-6, and CRP, is well documented. These markers are expressed at higher levels in patients with atherosclerosis compared to controls, as demonstrated by studies [50,51]. CRP, a key acute-phase protein associated with inflammatory and infectious processes, as well as tissue injury, serves as a reliable indicator of endothelial dysfunction in CAD [52].

One hypothesis regarding the link between *H. pylori* infection and CAD pathophysiology suggests that bacterial antigens can stimulate the growth of T and B lymphocytes and generate autoreactive antibodies through molecular mimicry. Matsuura et al. proposed that molecular mimicry involving *H. pylori* heat shock protein 60 (Hp-HSP60) may contribute to the pathogenesis of CAD by stimulating Th1 lymphocytes to produce IL-12 and INF-γ, or by activating macrophages crucial in the formation of atherosclerotic plaques [53].

Seropositivity for CagA is undeniably linked to heart disease, including unstable angina, atherosclerosis, cardiac X syndrome, and coronary artery disease, according to Mayr et al. [54]. Studies demonstrated that *H. pylori* infection increased the risk of acute coronary artery disease even in the absence of traditional risk factors [55]. The most plausible pathogenic mechanism linking *H. pylori* and CAD is the initiation of a chronic inflammatory process. *H. pylori* is hypothesized to play a unique role in CAD pathophysiology due to its ability to initiate a chronic, systemic, and persistent inflammatory state originating from the gastric epithelium, as noted by Mayr et al. [54].

*H. pylori* infection is associated with changes in the serum lipid profile and lipoprotein [a] levels, contributing to the risk of atherosclerosis and CVDs [45]. Persistent immune responses to *H. pylori*, including specific Th17 cells, may maintain the presence of an inflammatory state, even after the eradication of the infection, contributing to the risk of long-term complications [56].

In summary, *H. pylori* infection is associated with an increased risk of acute coronary artery disease and atherosclerosis through persistent inflammatory mechanisms and changes in lipid profiles. Inflammatory markers such as CRP and IL-6, linked to *H. pylori* infection, play significant roles in the development of atherosclerosis and CVDs [45,57,58]. PRRs, such as TLR4 and CD14, are crucial in subacute inflammation induced by *H. pylori*, influencing the susceptibility and severity of associated diseases [59,60]. Molecular mimicry by *H. pylori* antigens can stimulate autoimmune responses that significantly contribute to the development of atherosclerosis [54,61,62].

### 3.3. Myocardial Infarction and H. pylori

#### 3.3.1. Pathophysiological Mechanisms and Inflammation

*H. pylori* infection is closely associated with myocardial infarction through complex pathophysiological mechanisms. The primary virulence factor of *H. pylori*, CagA, is associated with an increased production of IL-8, a marker of inflammation. Contradictory findings exist regarding the correlation between *H. pylori* infection and atherosclerotic CAD. For example, Manolakis et al. found no link between *H. pylori* infection and CRP levels [63], while Carter et al. reported no link between *H. pylori* infection and the levels of fibrinogen or von Willebrand factor (vWF) [64]. Despite these findings, which suggest no association between *H. pylori* infection and certain inflammatory markers, the protein expression levels of CagA, a virulence factor of H. pylori, have been shown to be associated with an increased risk of myocardial infarction through inflammatory mechanisms.

*H. pylori* can induce systemic inflammation, increasing the risk of atherosclerosis and subsequently, myocardial infarction [65]. Infection with CagA-expressing *H. pylori* strains has been shown to be correlated with severe inflammation and immune cell activation in myocardial regions affected by infarction [66].

The significant inflammatory response induced by *H. pylori* infection, including IL-8 production, contributes to the pathogenesis of myocardial infarction. IL-8, a potent chemokine that attracts and activates neutrophils, plays a role in local and systemic inflammation [67]. In chronic *H. pylori* infections, elevated levels of IL-8 and TNF correlate with the severity of gastric inflammation and may contribute to the development of CVDs through systemic inflammatory mechanisms [68]. Inflammation caused by *H. pylori*, including the increase in IL-8 levels, is implicated in vascular wall damage and atherosclerosis progression, increasing the risk of myocardial infarction [69].

#### 3.3.2. Contradictions in the Literature

Numerous studies have examined the association between *H. pylori* infection and the risk of myocardial infarction, yielding often contradictory results. These inconsistencies reflect differing conclusions on the influence of the infection in CVDs. While certain studies have shown a moderate relationship between *H. pylori* infection and the risk of myocardial infarction, these associations are significantly reduced after adjusting for confounding factors, such as socioeconomic status and major cardiovascular risk factors [36].

Multiple studies examining seropositivity for the CagA antigen, a marker of more virulent *H. pylori* strains, suggest a modest association with coronary artery disease. However, these associations are also reduced after adjustment for socioeconomic factors [36]. A meta-analysis highlighted a small but significant association between vascular diseases and virulent *H. pylori* strains while finding no significant association with non-virulent strains [70]. Conversely, a prospective study [39] failed to demonstrate a strong association between *H. pylori* infection and the incidence of coronary heart disease, suggesting that the observed effects in earlier studies may result from confounding due to socioeconomic factors. Differences in the methodology and confounding factors, such as social status, smoking, and other cardiovascular risks, contribute to the variations among studies

Current studies provide mixed results regarding the association between *H. pylori* infection and the risk of myocardial infarction. Although certain studies suggest a modest relationship, this is often attenuated or absent after adjusting for socioeconomic confounding factors and other major risk factors. The differences in study design and the contribution of confounding factors may explain these discrepancies, highlighting the need for a well-designed large prospective multi-center study.

#### 3.3.3. Impact of *H. pylori* Eradication on the Risk of Myocardial Infarction and Mortality

Eradicating *H. pylori* may influence the risk of myocardial infarction by reducing the systemic inflammation associated with the infection. The STAMINA study demonstrated that antibiotic treatment for *H. pylori* significantly reduced adverse cardiac events in patients with acute coronary syndromes, independent of *H. pylori* seropositivity [71]. Similarly, another study found that *H. pylori* eradication significantly lowered the incidence of non-fatal myocardial infarction and readmission with angina in patients with acute coronary syndromes [72].

Comparing infected and uninfected individuals helps to elucidate the role of *H. pylori* in the pathogenesis of myocardial infarction. Observations indicated a higher prevalence of *H. pylori* infection among patients with acute myocardial infarction compared to the general population [73]. In Japanese patients < 55 years old, Kinjo et al. reported a significant association between *H. pylori* infection and increased the risk of myocardial infarction [24]. Another study found an inverse association between infection with CagA-positive *H. pylori* strains and cardiovascular mortality, suggesting a complex relationship between different bacterial strains and cardiovascular risk [74].

*H. pylori* infection has been linked to a higher incidence of acute coronary syndrome (ACS) in less-affluent countries compared to developed countries (OR 2.58 vs. 1.69), according to a comprehensive review and meta-analysis by Fang et al. [75]. Further research demonstrated an association between arterial stiffness, a predictor of cardiovascular events, and anti-*H. pylori* IgG antibodies by evaluating the cardio-ankle vascular index (CAVI). Those seropositive for *H. pylori* had significantly higher median values of age, systolic blood pressure, LDL-C levels, and median CAVI values [76].

In a large cohort of 198,487 Koreans receiving treatment for hypertension, *H. pylori* eradication was associated with a decreased risk of death from cerebrovascular disease, although not from congestive heart failure [77].

#### 3.3.4. Longitudinal Studies and the Long-Term Relationship between *H. pylori* and Myocardial Infarction

Research on the connection between myocardial infarction and *H. pylori* infection has yielded contradictory results. Ikeda et al. demonstrated that only CagA-positive *H. pylori* strains were associated with a higher risk of myocardial infarction [78]. In contrast, a meta-analysis involving 26,000 patients found a strong correlation between *H. pylori* infection and myocardial infarction risk [79], while another study reported a concomitant decrease in myocardial infarction and duodenal ulcers among individuals born between 1930 and 1980, indicating a potential temporal relationship between the decrease in myocardial infarction and *H. pylori* infection [36].

Due to these inconsistent findings, it is difficult to define *H. pylori* as a clear cause of CVDs. Longitudinal studies are essential to understand the relationship between *H. pylori* infection and long-term myocardial infarction incidence. For example, a prospective study by Whincup et al. found that *H. pylori* infection was associated with an increased risk of fatal myocardial infarction, but this association was significantly attenuated after adjusting for socioeconomic and other cardiovascular risk factors [80]. Similarly, the ARIC study, which investigated *H. pylori* seropositivity and coronary disease incidence over a median follow-up period of 3.3 years, found no significant association after adjusting for other risk factors, including fibrinogen and other inflammatory markers [28]. The Caerphilly study showed that *H. pylori* infection was initially associated with higher overall mortality and mortality as a result of fatal ischemic heart disease, but these associations lost statistical significance after adjusting for cardiovascular and socioeconomic risk factors [40].

Overall, while longitudinal studies suggest an association between *H. pylori* infection and myocardial infarction risk, this relationship is significantly influenced by socioeconomic factors and other cardiovascular risk factors, necessitating the need for rigorous adjustments to clarify the specific impact of *H. pylori*.

### 3.4. Stroke and H. pylori

#### 3.4.1. Pathophysiological Mechanisms

Stroke and *H. pylori* infection are closely linked through complex pathophysiological mechanisms. Chronic systemic inflammation induced by *H. pylori* may increase the risk of stroke via systemic inflammatory processes. Studies have indicated that chronic *H. pylori* infection is associated with systemic inflammation and an increased risk of stroke. Research highlighted that chronic *H. pylori* infection elevates the levels of inflammatory proteins and markers of endothelial dysfunction, suggesting a role in the development of atherosclerosis and CVDs [40]. Blum et al. demonstrated that eradicating *H. pylori* can improve endothelial dysfunction, indicating that this infection significantly contributes to endothelial impairment [41].

Infection with CagA-positive *H. pylori* strains has been associated with an increased risk of stroke and vascular dysfunction due to inflammatory effects and atherosclerotic plaque instability. Findings revealed that CagA-positive *H. pylori* strains are associated with carotid plaque instability and an increased risk of atherosclerotic stroke [81]. Diomedi et al. showed that CagA-positive *H. pylori* infection is associated with increased intima-media thickness and worse clinical outcomes in patients with atherosclerotic stroke [82].

*H. pylori* infection can lead to endothelial dysfunction, a key factor in cerebrovascular disease development. Studies demonstrated that *H. pylori* can significantly impair endothelial function through exosome-dependent mechanisms and the increased production of ROS, contributing to endothelial dysfunction and atherosclerosis [65]. Blum et al. observed that *H. pylori*-induced endothelial dysfunction can be reversed after the infection is eradicated, suggesting a direct link between the infection and endothelial impairment [41].

#### 3.4.2. Impact of *H. pylori* Infection on Platelets and Inflammatory Mediators

*H. pylori* can stimulate platelet activation and coagulation factors, contributing to thrombus formation and increased stroke risk. Research showed that *H. pylori* infection induced platelet aggregation through interaction with vWF and GPIb receptors on platelets, potentially leading to microvascular dysfunction and inflammatory cell recruitment [33]. Further studies highlighted that *H. pylori* caused persistent platelet activation in vivo by increasing lipid peroxidation and the release of 8-iso-prostaglandin F2α and 11-dehydro-thromboxane B2, a mechanism that may be involved in atherothrombosis [83]. Demonstrations indicated that *H. pylori*-produced urease activates platelets via a lipoxygenase-mediated pathway, contributing to the pathogenesis of cardiovascular and gastrointestinal diseases [84]. Findings revealed that the *H. pylori* VacA protein binds to multimerin 1 on platelets, inducing CD62P expression and platelet aggregation [85]. Elizalde et al. observed that *H. pylori* infection increases platelet aggregation and activation in infected patients, contributing to microvascular dysfunction and cellular inflammation [86].

*H. pylori* also increases the production of inflammatory mediators such as IL-6 and TNF-α, contributing to systemic inflammation and the risk of stroke. Research showed that *H. pylori* infection induced the production of pro-inflammatory cytokines, including IL-1β, IL-6, and TNF-α in gastric epithelial cells, contributing to gastric inflammation and potentially to systemic conditions such as stroke [87]. Studies demonstrated that urease produced by *H. pylori* is a potent stimulant of mononuclear phagocytes, inducing the production of inflammatory cytokines such as IL-1β and IL-6 [88]. Lindholm et al. observed that *H. pylori* infection increases IL-8, IL-1α, and IL-1β levels in gastric epithelial cells, which may contribute to the initial inflammatory response [89].

#### 3.4.3. Meta-Analyses and Cohort Studies on the Association between *H. pylori* and Stroke Risk

Meta-analyses have been used to assess data from multiple studies on the association between *H. pylori* and the risk of stroke. Findings indicated a significant association between chronic *H. pylori* infection and increased risk of ischemic stroke, particularly in non-cardioembolic stroke cases [90]. Gabrielli et al. showed that infection with CagA-positive *H. pylori* strains is associated with an increased risk of atherosclerotic stroke and carotid plaque instability [81]. Considerable evidence suggests that chronic *H. pylori* infection contributes to increased stroke risk through platelet activation mechanisms, the upregulation of inflammatory mediators, and endothelial dysfunction. The eradication of *H. pylori* may represent an effective strategy for reducing the risk of stroke.

Research suggests that *H. pylori* infection may be associated with elevated levels of inflammatory markers, which in turn may contribute to the risk of stroke. Brenner et al. showed that *H. pylori* infection is associated with increased levels of CRP and other inflammatory markers, contributing to chronic inflammation and the development of atherosclerosis [91]. Further research highlighted that *H. pylori* infection induced the production of pro-inflammatory cytokines, including IL-1β, IL-6, and TNF-α, contributing to systemic inflammation [87].

Regional variations in the association between *H. pylori* and stroke risk may be influenced by socioeconomic differences and access to treatment. Research showed that the prevalence of *H. pylori* infection and its association with stroke can vary significantly between different geographic regions and socioeconomic groups. For example, in Japan, *H. pylori* infection was not significantly associated with the risk of stroke, but there was a trend toward an association between CagA-positive *H. pylori* infection and myocardial infarction risk [78]. Jiang et al. evaluated the population-attributable burden for gastric cancer, coronary heart disease, and stroke in China, concluding that *H. pylori* infection is responsible for a significant percentage of ischemic stroke cases, highlighting the importance of infection eradication for reducing chronic diseases [92]. Consistent evidence suggests that chronic *H. pylori* infection is associated with elevated levels of inflammatory markers, contributing to the risk of stroke. Additionally, regional variations in the association between *H. pylori* and the risk of stroke underscore the importance of socioeconomic differences and access to treatment in understanding this link.

### 3.5. Endothelial Dysfunction and H. pylori

#### 3.5.1. Inflammatory and Immune Mechanisms

Chronic inflammation induced by *H. pylori* plays a crucial role in endothelial dysfunction, a critical process in the pathogenesis of CVDs. Studies have shown that chronic *H. pylori* infection is associated with elevated levels of CRP and soluble intercellular adhesion molecule-1 (ICAM-1), which correlate with reduced flow-mediated vasodilation [40]. Xia et al. demonstrated that *H. pylori* infection increases oxidative stress and reduces nitric oxide (NO) production, contributing to vascular endothelial dysfunction and atherosclerosis [43].

#### 3.5.2. Direct Interaction between *H. pylori* and Endothelial Cells

*H. pylori* directly acts on human endothelial cells, resulting in an increase in the expression of adhesion molecules such as VCAM-1, ICAM-1, and E-selectin [93]. This activation leads to increased neutrophil recruitment and tissue damage. The type IV secretion system (T4SS) of *H. pylori* and its adhesin subunit, CagL, play crucial roles in inflammatory responses. Tafreshi et al. demonstrated that these components significantly increase IL-6 and IL-8 levels in endothelial cells, highlighting a specific pathway through which *H. pylori* triggers endothelial inflammation [94].

#### 3.5.3. Role of Inflammatory Cytokines in *H. pylori*-Induced Endothelial Dysfunction

Inflammatory cytokines, such as IL-6 and TNF-α, are upregulated in *H. pylori* infections, leading to endothelial dysfunction, and studies found that these cytokines contribute to oxidative stress and reduce NO availability, critical factors in endothelial impairment [95]. *H. pylori* infection increases IL-8 secretion in endothelial cells, promoting chemotaxis and inflammation, and Rasmi et al. showed that this response is particularly significant in the context of CagA+ *H. pylori* strains [96].

#### 3.5.4. Mechanisms of PRR Activation by *H. pylori*

*H. pylori* infection and PRR activation involve the recognition by PRRs, contributing to inflammation and endothelial dysfunction. Research demonstrated that TLR2 and TLR4 are essential in *H. pylori* recognition, inducing the formation of TLR2/1/CD36 complexes, and triggering inflammatory responses [97]. Studies showed that interaction with TLRs triggers the production of pro-inflammatory cytokines. *H. pylori* is recognized by TLRs (TLR2, TLR4, TLR5, and TLR9), activating downstream signaling pathways leading to the production of inflammatory mediators through the activation of NF-κB, MAP kinases, and IRF signaling pathways [98]. Pachathundikandi et al. observed that *H. pylori* can evade recognition by TLR5 due to a conserved N-terminal motif of flagellin [99].

#### 3.5.5. Oxidative Stress and Endothelial Dysfunction in the context of *H. pylori* Infection

Oxidative stress plays a crucial role in endothelial dysfunction associated with *H. pylori* infection. ROS generated during infection contributes to endothelial damage and inflammatory processes. Studies showed that *H. pylori* induced the production of IL-1β and IL-18 through the activation of the NLRP3 inflammasome [100]. Infection with *H. pylori* in THP-1 cells resulted in an increased production of ROS, which activates the NLRP3 inflammasome, contributing to the secretion of pro-inflammatory cytokines. Research highlighted the protective role of the antioxidant enzyme PRDX2 against *H. pylori*-induced oxidative stress, underscoring the importance of ROS regulation in the context of infection [101]. Radulescu et al. emphasized the importance of physical exercise and dietary adaptations, such as a ketogenic diet, in increasing resilience against oxidative stress [102].

#### 3.5.6. Involvement of Endothelial Adhesion Molecules in *H. pylori*-Induced Vascular Inflammation

*H. pylori* affects the expression of endothelial adhesion molecules, contributing to vascular inflammation and endothelial dysfunction. Observations indicated that *H. pylori* infection increased TLR4 expression in the gastric mucosa, and this was correlated with neutrophil infiltration and mucosal inflammation [103]. Cotran et al. demonstrated that *H. pylori* activated endothelial activation antigen in vivo, leading to its expression in delayed hypersensitivity reactions and pathological tissues [104].

*H. pylori* infection contributes to endothelial dysfunction through chronic inflammation, direct bacterial interactions with endothelial cells, and the action of inflammatory cytokines. These mechanisms collectively lead to oxidative stress, reduced NO availability, and increased expression of adhesion molecules, which are critical in the pathogenesis of CVDs.

### 3.6. Controversial Findings Regarding the Causal Link between H. pylori and CVDs

#### 3.6.1. Discrepancies in the Results from Epidemiological Studies

There is considerable disagreement in the results of studies assessing a causal link between *H. pylori* infection and CVDs. Certain studies have suggested that *H. pylori* is not a risk factor for CVDs, while others support an inverse relationship between infection with CagA-positive *H. pylori* strains and cardiovascular mortality. For example, Schöttker et al. observed an inverse relationship [105], whereas Lin et al. found no significant association between *H. pylori* and cardiovascular mortality [39]. The results of epidemiological studies are often contradictory. Whincup et al. found an association between *H. pylori* infection and the risk of myocardial infarction and stroke, but this association was substantially reduced after adjusting for confounding factors [35]. Other research showed that *H. pylori* infection is not associated with cardiovascular risk factors, such as hypertension or lipid profiles [14].

#### 3.6.2. Confounding Factors in Research on *H. pylori* and CVDs

Confounding factors such as socioeconomic status, smoking, and other comorbidities can influence the apparent relationship between *H. pylori* and CVDs. For example, Strachan et al. demonstrated that, in studies controlling for these factors, the association between *H. pylori* and CVDs was often reduced or eliminated [28]. Future research should focus on elucidating the mechanisms by which *H. pylori* may contribute to the pathogenesis of CVDs. Advanced and well-defined methodological approaches and technologies are necessary to better understand the complex relationships between *H. pylori* infection, inflammatory response, and endothelial dysfunction. Longitudinal and interventional studies may provide valuable insights into the potential benefits of *H. pylori* eradication in preventing CVDs. In conclusion, the causal relationship between *H. pylori* and CVDs remains contested and requires further investigation to clarify the roles of confounding factors and the specific pathogenic mechanisms involved.

#### 3.6.3. Impact of *H. pylori* Eradication on CVDs

The impact of *H. pylori* eradication on cardiovascular risk is variable, with mixed results reported in different studies. Research showed that *H. pylori* eradication can lead to changes in cardiovascular risk factors, including significant increases in body weight, body mass index, total cholesterol, LDL, triglycerides, and γ-GTP during the first and second years after eradication [106]. A meta-analysis indicated that *H. pylori* eradication is associated with changes in the lipid profile, leading to increased HDL cholesterol and triglyceride levels, but without a significant impact on LDL cholesterol [107].

The eradication of *H. pylori* and the risk of coronary heart disease (CHD) has been the subject of numerous studies. For example, findings reported a positive association between *H. pylori* eradication and a reduced risk of CHD, showing that patients who received *H. pylori* eradication within the first year of diagnosis had a lower risk of CHD compared to those who were not treated [108]. However, *H. pylori* eradication does not have a significant effect on metabolic and inflammatory parameters, as shown by research that did not observe significant changes in blood glucose, lipid profile, insulin resistance, white blood cell count, and CRP after eradication [109].

#### 3.6.4. Investigating Potential Targeted Therapies for *H. pylori* in the Context of CVDs

Targeted therapy for *H. pylori* infection and its impact on CVDs represents an important research direction. Findings demonstrated that *H. pylori* eradication reduces arterial lumen loss after percutaneous coronary angioplasty, suggesting that eradication may mitigate chronic inflammation and the release of pro-inflammatory cytokines [110]. Additionally, Chen et al. highlighted that probiotic supplementation after *H. pylori* eradication can improve gastrointestinal symptoms and help restore gut microbiota balance [111].

In conclusion, the eradication of *H. pylori* has a variable impact on CVDs, with some studies indicating benefits in reducing the risk of CHD and positive changes to the lipid profile, while others did not observe significant changes in the metabolic and inflammatory parameters. Targeted therapy and the use of probiotics may represent promising directions for future research.

## 4. Neurological Diseases

### 4.1. Neurological Diseases and H. pylori Infection

*H. pylori*, a Gram-negative bacterium colonizing the stomach, is well-known for its role in several gastrointestinal diseases, such as gastric and duodenal ulcers [112]. Recent studies suggest a link between this infection and various neurological conditions [113]. Research has indicated that the chronic inflammation and immune response induced by *H. pylori* may contribute to the development and progression of neurological diseases, leading to neuroinflammation and neuronal dysfunction [114].

### 4.2. Prevalence of H. pylori Infection in Neurological Disease Populations

Epidemiological studies have provided data on the prevalence of *H. pylori* infection in various populations affected by neurological diseases. Findings showed that *H. pylori* infection is present in a significant percentage of patients with severe neurological conditions, including those hospitalized for gastrointestinal symptom evaluation [115]. Similarly, reports indicated a high prevalence of *H. pylori* infection in patients with Parkinson’s disease and other neurological disorders, suggesting a link between *H. pylori* colonization and these conditions [116].

### 4.3. The Link between H. pylori and Various Neurological Conditions

Chronic inflammation induced by *H. pylori* infection may contribute to the development of neurological diseases through pro-inflammatory mediators that can reach the brain, causing neuronal inflammation. This has been associated with diseases such as Parkinson’s and Alzheimer’s [16]. Changes in the gut microbiota influenced by *H. pylori* infection can affect the central nervous system through the gut–brain axis, contributing to neurological disorders such as multiple sclerosis and Parkinson’s disease [117]. Various epidemiological studies have demonstrated a correlation between *H. pylori* infection and neurological disorders, emphasizing the importance of eradicating the infection to reduce the risk of developing these conditions [118].

### 4.4. Risk Factors Associated with H. pylori Infection and Neurological Diseases

Systemic chronic inflammation induced by *H. pylori* can affect central nervous system functioning, contributing to the development of neurological diseases through the release of pro-inflammatory cytokines and other vasoactive substances [119]. *H. pylori* infection may influence the absorption of essential medications for treating neurological diseases and can lead to micronutrient deficiencies, exacerbating symptoms [120]. Kountouras et al. showed that *H. pylori* infection triggered immune reactions due to molecular mimicry, where bacterial components similar to host antigens cause an autoimmune reaction, influencing the progression of neurological disorders [117].

### 4.5. Socioeconomic and Geographic Impact on the Prevalence of H. pylori in Neurological Diseases

Socioeconomic factors play a significant role in the prevalence of *H. pylori* infection, including in neurological diseases. Lower education levels, crowded living conditions, and limited access to medical care are associated with higher infection rates [121]. Occupation and work environment can also influence the prevalence of *H. pylori* infection. For example, agricultural and healthcare workers have higher infection rates due to increased exposure to pathogens [122]. Geographic variations in *H. pylori* prevalence are determined by differences in sanitary conditions, dietary habits, and access to medical treatments [123].

### 4.6. Proposed Pathogenic Mechanisms Linking H. pylori and Neurological Diseases

Systemic inflammation and autoimmunity are the primary pathogenic mechanisms proposed for the link between *H. pylori* infection and neurological diseases. Chronic *H. pylori* infection induces a systemic inflammatory response, with the secretion of pro-inflammatory cytokines that can affect the brain [16]. Molecular mimicry is another proposed mechanism, where *H. pylori* antigens are similar to host tissue antigens, leading to an autoimmune response that may contribute to the development of neurological diseases [19].

### 4.7. Systematic Reviews and Meta-Analyses on H. pylori and Neurological Diseases

Recent systematic reviews and meta-analyses have found that *H. pylori* infection is associated with several neurological diseases, including Alzheimer’s disease and multiple sclerosis [123]. Another meta-analysis indicated that, while *H. pylori* infection might have a protective effect against certain digestive diseases, its effects on neurological diseases require further research [124].

### 4.8. Alzheimer’s Disease and H. pylori

#### 4.8.1. Pathophysiological Mechanisms and Inflammation

Evidence shows that individuals with an *H. pylori* infection have a 1.6-fold increased risk of developing Alzheimer’s disease compared to those without an infection, indicating a potential role of *H. pylori* in Alzheimer’s disease pathogenesis [125]. It has been demonstrated that the apolipoprotein E (ApoE) 4 polymorphism was more common in patients with Alzheimer’s disease and *H. pylori* infection than in uninfected patients [126]. The ApoE 4 polymorphism is the strongest genetic risk factor for Alzheimer’s disease [127]. Cerebrospinal fluid [CSF] and serum from patients with Alzheimer’s disease showed significantly higher levels of specific anti-*H. pylori* antibodies (anti-*H. pylori* IgG) compared to those with normal cognition of the same age [128].

*H. pylori* infection can induce chronic systemic inflammation, potentially contributing to neuroinflammation and the development of Alzheimer’s disease. Animal model studies have shown that *H. pylori* infection leads to severe gastritis and increased neuroinflammation, although without amyloid deposits in the brain [129]. *H. pylori* may induce an irregular immune response that, through molecular mimicry, contributes to neuronal damage and the pathogenesis of Alzheimer’s disease [114]. *H. pylori* infection triggers the release of pro-inflammatory factors such as cytokines (IL-1β and TNF-α) and chemokines, which can enter the circulation and affect the central nervous system, contributing to neurodegeneration [16].

#### 4.8.2. ApoE Polymorphisms and the Immune Response

Studies suggest that the ApoE4 polymorphism may be associated with increased susceptibility to *H. pylori* infection and may influence the severity of Alzheimer’s disease. For example, the ApoE4 protein may facilitate the entry of pathogens, including *H. pylori*, into the brain [130]. Elevated levels of specific anti-*H. pylori* antibodies (IgG) in the CSF and serum of patients with Alzheimer’s disease indicate a possible involvement of this infection in disease pathogenesis. The antibody levels in the CSF correlate with disease severity [114].

*H. pylori* can affect the blood–brain barrier [BBB] by inducing systemic inflammation and releasing bacterial toxins that can cross the barrier, contributing to neurodegeneration [131]. Pro-inflammatory cytokines, such as TNF-α, and matrix metalloproteinases [MMPs] can disrupt BBB integrity, facilitating *H. pylori* entry into the brain and exacerbating neuroinflammation and neurodegeneration [132]. It has been hypothesized that *H. pylori* may access the central nervous system via the oral–nasal–olfactory route or through the gastrointestinal tract, contributing to neurodegenerative processes involved in Alzheimer’s disease [133].

#### 4.8.3. Relevant Studies and Contradictory Theories

There are numerous studies on the relationship between *H. pylori* and dementia. Evidence found that infected individuals had a 1.5-fold higher incidence of dementia compared to uninfected individuals over a 20-year follow-up period [131]. Beydoun et al. also identified a link between *H. pylori* infection and cognitive decline [134]. Additionally, a study by Chang et al. has shown that eradicating *H. pylori* infection can have a favorable impact on the progression of Alzheimer’s disease in patients with peptic ulcers, demonstrating a decreased risk of dementia progression compared to those who did not undergo eradication therapy [135]. One explanation for the correlation between *H. pylori* and Alzheimer’s disease is that the bacteria may enter the brain via the oral–nasal–olfactory route, leading to neurodegeneration. The “Trojan horse” theory proposes that *H. pylori* enters the brain through infected monocytes, disrupting the BBB and increasing the production of inflammatory mediators such as TNF-α, which can further disrupt the BBB by upregulating MMPs [136]. Another theory posits that *H. pylori* can enter the brain through the gastrointestinal tract and induce neurodegeneration via a rapid retrograde neuronal pathway [137]. A Japanese study by Yamada et al. did not support the hypothesis that *H. pylori* infection causes Alzheimer’s disease [138]. The discrepancy with research conducted in Western countries, where the infection is less common, may be partially explained by the high prevalence of *H. pylori* in controls. Additionally, research showed that eliminating the infection slowed the progression of the neurological condition, supporting the notion that *H. pylori* may play a role in Alzheimer’s disease [135].

Studies suggest that the prevalence of *H. pylori* infection and the risk of Alzheimer’s disease varies significantly across different regions and cultures, influenced by factors such as sanitary conditions and dietary habits [139]. The role of *H. pylori* infection in the development of Alzheimer’s disease is highly contested, with studies supporting both positive and negative links, highlighting the need for further research to clarify these associations [136].

There is consistent evidence suggesting a link between *H. pylori* infection and cognitive decline, but the causal relationship remains unclear. The eradication of the infection may have benefits for the symptoms of Alzheimer’s disease, but further studies are necessary to fully understand these associations.

### 4.9. Multiple Sclerosis (MS) and H. pylori

#### 4.9.1. Pathophysiological Mechanisms

The introduction of the SS1 antigen of *H. pylori* into an animal model of MS, known as experimental autoimmune encephalomyelitis (EAE), suggests that *H. pylori* has immunomodulatory capabilities that may impact the pathogenesis of MS [140]. Due to the molecular similarity between AQP4 and bacterial AQP, *H. pylori* infection appears to be a risk factor for the production of anti-AQP4 antibodies in MS [141]. Furthermore, Cook et al. showed that, by suppressing both Th1 and Th17 responses, *H. pylori* may have a protective effect against EAE [142].

#### 4.9.2. *H. pylori*-Induced Immunomodulation in the Context of Multiple Sclerosis

The immunomodulatory properties of *H. pylori* are highlighted in a study on animal models, which showed that *H. pylori* infection can reduce disease severity in EAE [142]. *H. pylori* infection inhibited Th1 and Th17 responses, suggesting a protective effect through the suppression of these immune responses. Research indicates that the seroprevalence of *H. pylori* is significantly lower among patients with MS compared to the healthy controls, suggesting a possible protective effect of *H. pylori* infection against the development of MS [143].

#### 4.9.3. SS1 Antigen and Animal Models of MS

The impact of the SS1 antigen in experimental models of MS was investigated in a study, which showed that systemic immunization with the *H. pylori* SS1 antigen can modulate immune responses affecting MS [140]. Another study demonstrated that there was an immunological reaction against the AQP4 protein in patients with MS and neuromyelitis optica (NMO) who were seropositive for *H. pylori*, suggesting that *H. pylori* antigens can trigger autoimmunity through molecular mimicry, contributing to the pathogenesis of these diseases [144]. The reduction in these immune responses may explain the observed protective effect of *H. pylori* infection in MS.

#### 4.9.4. Negative Correlation between *H. pylori* and MS

A meta-analysis conducted by Jaruvongvanich et al. showed a significantly lower prevalence of *H. pylori* infection among patients with MS compared to healthy controls, suggesting a possible protective effect of the infection against the development of MS [143]. Studies have shown that *H. pylori* infection is more frequent in patients with MS who present with anti-AQP4 antibodies, a specific marker for NMO [144]. There is evidence suggesting that *H. pylori* infection may have an immunomodulatory effect that can influence the development and progression of MS by suppressing Th1 and Th17 responses [145]. There is evidence suggesting a possible protective effect of *H. pylori* infection against MS, but the exact mechanisms and relationship with other autoimmune conditions, such as NMO, require further research [144].

#### 4.9.5. Epidemiological and Cohort Studies on *H. pylori* and Multiple Sclerosis

A meta-analysis of six observational studies involving 1902 participants showed a significantly lower prevalence of *H. pylori* infection among patients with MS compared to healthy controls, suggesting a possible protective effect of *H. pylori* against MS [146]. An experimental study on an animal model of MS showed that *H. pylori* infection in mice reduced the severity of EAE, by inhibiting Th1 and Th17 responses [142]. A cohort study in Japan showed that seropositivity for *H. pylori* was significantly lower in patients with conventional MS compared to patients with opticospinal MS and healthy controls, suggesting a specific protective effect of *H. pylori* against conventional MS [144].

#### 4.9.6. The Role of *H. pylori* in the Etiology of MS

Studies show contradictory results regarding the role of *H. pylori* infection in the development of MS. Certain studies suggest a protective effect, while others have not found a significant correlation. An updated review concluded that *H. pylori* may have a protective role, but more research is needed to clarify this relationship [146]. Research by Ahadiat et al. showed a lower prevalence of *H. pylori* infection in patients with MS compared to healthy controls, suggesting a possible protective effect of *H. pylori* against MS [147].

#### 4.9.7. Impact of *H. pylori* Eradication on Patients with MS

Studies have shown that the eradication of *H. pylori* does not have a significant impact on the progression of MS, suggesting that the possible protective effects of *H. pylori* infection are not clearly influenced by eradication [148]. A study by Ranjbar et al. showed that patients with MS who are seropositive for *H. pylori* have lower levels of pro-inflammatory cytokines (IFN-γ, TNF-α, IL-6, and IL-17) and higher levels of anti-inflammatory cytokines (IL-4 and IL-10) compared to seronegative patients, suggesting an immunomodulatory effect of *H. pylori* on MS [145].

In conclusion, there is evidence suggesting that *H. pylori* infection may have a protective effect against multiple sclerosis through immunomodulatory mechanisms. However, the results are contradictory, and further research is required to fully understand this relationship and to determine the impact of *H. pylori* eradication on the course of the disease.

### 4.10. Parkinson’s Disease and H. pylori

#### 4.10.1. Pathophysiological Mechanisms

The degeneration of dopaminergic neurons in the basal ganglia system of the substantia nigra pars compacta underlies Parkinson’s disease. Chronic gastrointestinal disorders are linked to pro-inflammatory cytokines that can disrupt the BBB, leading to brain inflammation and dopaminergic neuronal death, potentially resulting in parkinsonism [16,149,150]. Studies have shown that systemic inflammation can compromise BBB integrity, allowing inflammatory molecules and cells to enter the brain. This may lead to brain inflammation and neurodegeneration in diseases such as Parkinson’s disease [149]. Microglia, the brain’s resident immune cells, migrate to cerebral vessels during systemic inflammation and initially maintain BBB integrity. However, sustained inflammation causes microglia to degrade the endothelial component of the BBB, impairing its function [150]. *H. pylori* infection induces the secretion of pro-inflammatory cytokines, such as TNF-α and IL-6, which can cross a compromised BBB and contribute to dopaminergic neuronal death, promoting parkinsonism [16]. Chronic *H. pylori* infection induces a persistent systemic inflammatory response that can damage the BBB and lead to chronic brain inflammation, contributing to the neurodegeneration observed in Parkinson’s disease. There is a link between chronic gastrointestinal disorders caused by *H. pylori* infection and brain inflammation, suggesting that systemic inflammation can mediate pathological effects in the brain, contributing to Parkinson’s disease [151]. *H. pylori* infection can induce neurodegeneration through mechanisms involving systemic inflammation and BBB impairment. Studies indicate that *H. pylori* may contribute to the accumulation of neurotoxic factors in the brain, accelerating dopaminergic neuronal loss, according to Baudron et al. [113].

#### 4.10.2. Relevant Studies

A 2017 meta-analysis evaluated eight relevant studies involving 33,125 individuals, indicating a potential association between *H. pylori* infection and the risk of Parkinson’s disease [152]. A recent study in the general population of Taiwan found a significant correlation between *H. pylori* infection and an increased risk of Parkinson’s disease among those aged ≥60 years old, but not among younger individuals. L-3,4-dihydroxyphenylalanine (L-dopa), which is used to treat Parkinson’s disease, may have its bioavailability affected by *H. pylori* infection as it can disrupt the duodenal mucosa, where L-dopa is absorbed [151]. Better clinical outcomes and increased L-dopa absorption seem to be linked to the eradication of the infection [153]. A meta-analysis demonstrated that *H. pylori* infection is associated with an increased risk of Parkinson’s disease, with a combined odds ratio of 1.59 [152]. Another meta-analytical study showed that *H. pylori* infection can exacerbate the symptoms of Parkinson’s disease and that eradicating the infection can improve the clinical condition of patients [120]. A retrospective cohort study analyzed health insurance data from Taiwan found that *H. pylori* infection is associated with an increased risk of Parkinson’s disease, with an adjusted hazard ratio of 2.29 [151]. *H. pylori* infection can reduce L-dopa bioavailability by affecting its absorption, which may exacerbate the motor symptoms of Parkinson’s disease [153]. *H. pylori* eradication treatment has been associated with significant improvements in motor symptoms in patients with Parkinson’s disease, including reduced daily “off time” and increased daily “on time” [154]. Studies have shown that patients with Parkinson’s disease with *H. pylori* infection exhibit higher levels of pro-inflammatory cytokines, such as TNF-α and IL-6, which may contribute to disease progression [155].

In conclusion, meta-analyses and cohort studies indicate a significant link between *H. pylori* infection and an increased risk of Parkinson’s disease. *H. pylori* eradication treatment may improve motor symptoms in patients. A deeper understanding of the biological mechanisms underlying this association is needed.

### 4.11. Guillain–Barré Syndrome (GBS) and H. pylori

#### 4.11.1. The Pathophysiological Mechanisms of GBS and the Role of *H. pylori*

GBS is an acute autoimmune neuropathy characterized by progressive distal-to-proximal limb paralysis, initially affecting the arms and legs. The involvement of the autonomic nervous system or respiratory muscles can lead to potentially fatal consequences. Studies have indicated that *H. pylori* infection may be associated with the development of GBS through molecular mimicry mechanisms between bacterial antigens and peripheral nervous system structures [120]. Additionally, antibodies against *H. pylori* proteins, including HSP and UB proteins, have been detected in the CSF of patients with GBS, suggesting an autoimmune response induced by structural similarity [156]. Other studies have shown that molecular mimicry between ganglioside-like structures on the bacterial surface and human gangliosides contributes to GBS pathogenesis [157].

*H. pylori* infection can trigger an autoimmune response leading to demyelination and neuropathy. Annunziata et al. detected antibodies against *H. pylori* in the serum and CSF of patients with GBS [158]. Specific antibodies against the VacA toxin produced by *H. pylori* have been found in the CSF of patients with GBS, suggesting the involvement of this toxin in the pathogenesis of this disease through direct damage to Schwann cells and demyelination [156].

#### 4.11.2. Relevant Studies on *H. pylori* Infection and GBS

Several studies have indicated that patients with GBS had significantly higher serum levels of anti-*H. pylori* IgG compared to controls; 20% of the controls and 80% of the patients had positive levels of anti-*H. pylori* IgG in the CSF [120]. IgG antibodies against *H. pylori* cytotoxin-associated gene A (VacA) were found in the CSF of patients with GBS [156]. The limited sample sizes in these investigations are a limitation; thus, larger samples are required to establish a true cause–effect association between *H. pylori* and GBS. A meta-analysis highlighted increasing evidence suggesting the involvement of *H. pylori* infection in GBS development, consolidating findings from multiple studies [120].

*H. pylori* infection was more frequent in patients with acute inflammatory demyelinating polyradiculoneuropathy compared to the control group. This indicates an association between *H. pylori* infection and the demyelinating subtype of GBS [159]. Specific antibodies against the VacA toxin produced by *H. pylori* were detected in the CSF of patients with GBS, suggesting that VacA may play a role in GBS pathogenesis through antibody-induced demyelination [156]. Studies show a significant prevalence of anti-*H. pylori* antibodies in patients with GBS, suggesting a potential pathogenic role of this bacterium in GBS development. *H. pylori* infection and its VacA toxin are associated with immune responses that may contribute to demyelination and neuropathy.

#### 4.11.3. Clinical and Demographic Considerations

Research indicates that the prevalence and clinical presentation of GBS can vary significantly depending on different regions and demographics. For example, incidence rates and clinical outcomes in regions such as Europe, Asia, and the Americas show distinct patterns, likely influenced by the prevalence of *H. pylori* and other antecedent infections [160]. Although specific studies on the direct impact of *H. pylori* eradication on GBS outcomes are limited, certain research suggests that addressing *H. pylori* infection could influence the disease course. For example, treating associated infections in patients with GBS may affect recovery and prognosis [158]. Observations from various clinical settings emphasize the importance of managing infections in patients with GBS. It has been shown that addressing infections, such as *H. pylori*, can be part of a broader GBS management strategy, although more specific research is required to confirm these effects [114].

In conclusion, there is substantial evidence linking *H. pylori* infection to GBS, highlighting the need for further research to understand how managing this infection could influence GBS outcomes. Geographic and demographic variations in GBS incidence and presentation underscore the complexity of this relationship and the importance of region-specific studies.

### 4.12. Discussions on Controversies and Future Research Proposals Regarding Neurological Diseases and H. pylori

#### 4.12.1. Stroke and *H. pylori*

There are conflicting results in the literature regarding the link between *H. pylori* infection and the risk of stroke. For example, one study reported that *H. pylori* infection is not associated with increased mortality among stroke patients [36]. In contrast, a recent meta-analysis indicated that the presence of CagA-positive *H. pylori* strains and persistent *H. pylori* infection are significant risk factors for ischemic stroke [28].

Detailed mechanistic studies and clinical trials are needed to clarify how *H. pylori* infection may contribute to the pathogenesis of stroke and to investigate whether *H. pylori* eradication treatments can influence disease progression.

#### 4.12.2. Alzheimer’s Disease and *H. pylori*

There are contradictory findings regarding the role of *H. pylori* infection in Alzheimer’s disease. Some researchers have demonstrated an association between *H. pylori* infection and the exacerbation of neurodegenerative inflammation, increasing the risk of Alzheimer’s disease [117]. On the other hand, other studies have found no significant link [148].

Detailed mechanistic studies are needed to understand how *H. pylori* infection may contribute to the pathogenesis of Alzheimer’s disease and to investigate whether *H. pylori* eradication treatments can influence disease progression.

#### 4.12.3. MS and *H. pylori*

The current literature shows contradictory results regarding the influence of *H. pylori* infection on multiple sclerosis [MS]. Some studies suggest that *H. pylori* infection may have a protective effect due to immune response modulation, while others have found no clear association [145].

Future research should focus on investigating how *H. pylori* infection affects the immune response in patients with MS and whether eradicating the infection can influence the disease course.

#### 4.12.4. Parkinson’s Disease and *H. pylori*

There is conflicting evidence regarding the association between *H. pylori* infection and Parkinson’s disease. Some studies indicate a possible link [118], while others find no significant association. There are also discrepancies regarding the proposed pathophysiological mechanisms, including chronic inflammation and autoimmune responses [116].

Future research should focus on longitudinal studies to clarify the causal relationship and assess the impact of anti-*H. pylori* treatment on the progression of Parkinson’s disease. Detailed molecular and immunological studies are needed to understand the exact mechanisms by which *H. pylori* can influence the development of Parkinson’s disease.

#### 4.12.5. GBS and *H. pylori*

There are conflicting reports on the role of *H. pylori* in triggering Guillain–Barré syndrome [GBS]. Some studies suggest a possible link [126], while others have found no significant association [120]. Investigations into pathogenic mechanisms, including molecular mimicry and immune response, provide mixed and insufficient results to draw a clear conclusion.

Future research should include larger, well-controlled studies to clarify the prevalence of anti-*H. pylori* antibodies in the serum and cerebrospinal fluid (CSF) of patients with GBS compared to healthy controls. A detailed investigation of the molecular and immunological mechanisms by which *H. pylori* contributes to the pathogenesis of GBS is necessary, including experimental and clinical studies.

## 5. Discussion

*H. pylori*, a Gram-negative bacterium colonizing the stomach, is well-known for its role in various gastrointestinal diseases. Recent research has highlighted a connection between this infection and the development of cardiovascular and neurological conditions. This discussion will analyze the pathophysiological mechanisms involved in myocardial infarction, Alzheimer’s disease, and multiple sclerosis, integrating the role of *H. pylori* infection in these processes.

### 5.1. Cardiovascular Diseases

**Figure 1:**
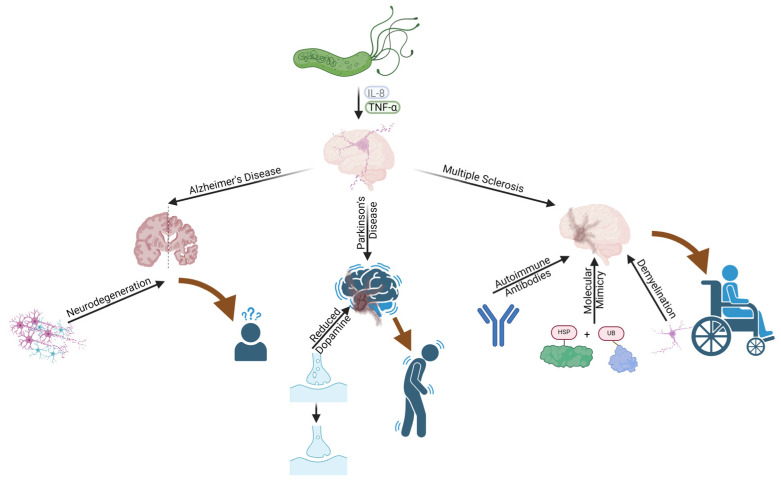
Relationship between *H. pylori* infection and neurological diseases—created with BioRender.com.

Atherosclerosis is characterized by the formation of atherosclerotic plaques in arteries, leading to their narrowing and an increased risk of vascular obstruction. The process begins with endothelial dysfunction and chronic inflammation, promoting lipid accumulation and plaque formation. *H. pylori* infection induces chronic and systemic inflammation through the release of pro-inflammatory cytokines (IL-8, TNF-α) (Figure 1). This contributes to endothelial dysfunction and atherosclerotic plaque formation, increasing the risk of vascular obstruction and, consequently, cardiovascular diseases [20,21,22,23].

Myocardial infarction occurs when blood flow to a part of the heart muscle is blocked, usually due to a thrombus formed on a ruptured atherosclerotic plaque. This leads to cardiac tissue death due to lack of oxygen. Chronic inflammation and endothelial dysfunction caused by *H. pylori* infection can favor plaque formation and rupture, as well as thrombosis, increasing the risk of myocardial infarction. Studies have shown that patients with *H. pylori* have higher levels of inflammatory markers and an increased cardiovascular risk [12,24].

Ischemic stroke occurs when a blood vessel in the brain is blocked, leading to brain tissue death due to lack of oxygen. Endothelial dysfunction and inflammation play crucial roles in developing this condition. *H. pylori* infection can exacerbate endothelial dysfunction and systemic inflammation, contributing to thrombus formation and vascular occlusions, increasing the risk of ischemic stroke [12,29,31].

### 5.2. Neurological Disorders

**Figure 2:**
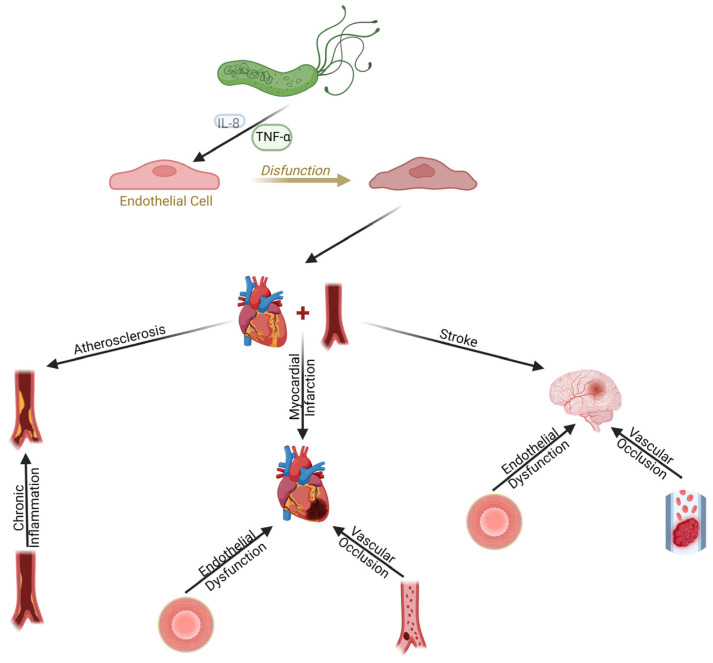
Relationship between *H. pylori* infection and cardiovascular diseases—created with BioRender.com.

Alzheimer’s disease is characterized by progressive neurodegeneration, the accumulation of beta-amyloid plaques, and tau neurofibrillary tangles in the brain, leading to memory loss and cognitive decline (Figure 2). Chronic inflammation induced by *H. pylori* can contribute to neuroinflammation and blood–brain barrier [BBB] dysfunction, allowing neurotoxic factors to enter the brain and worsen neurodegeneration. Studies have indicated a higher prevalence of *H. pylori* infection in Alzheimer’s patients, suggesting a possible role of the bacterium in the disease’s pathogenesis [16,117].

Parkinson’s disease is characterized by the loss of dopaminergic neurons in the substantia nigra, leading to motor symptoms such as tremor, rigidity, and bradykinesia. Systemic inflammation and BBB dysfunction caused by *H. pylori* can allow pro-inflammatory cytokines to reach the brain, contributing to dopaminergic neuron death. Studies have shown that eradicating *H. pylori* may improve motor symptoms in Parkinson’s patients, indicating a link between infection and disease pathogenesis [151,152].

MS is an autoimmune disease where the immune system attacks the myelin sheath covering nerve fibers, leading to nerve damage and various neurological symptoms. *H. pylori* infection may induce an autoimmune response through molecular mimicry, where bacterial antigens similar to host antigens trigger an autoimmune attack on myelin. Studies suggest an immunomodulatory effect of *H. pylori*, which may protect against MS by suppressing Th1 and Th17 responses [144,145].

### 5.3. Clinical Relevance

The relevance of *H. pylori* infection in developing cardiovascular and neurological diseases is significant for clinical practice, influencing therapeutic approaches and treatment strategies. Eradicating *H. pylori* infection can offer major benefits, not only in reducing the risk of gastrointestinal diseases but also in ameliorating symptoms and lowering the risk of cardiovascular and neurological complications.

#### 5.3.1. Therapies for Cardiovascular Diseases

Therapeutic approaches for cardiovascular diseases include using antibiotics to eradicate *H. pylori*, combined with anti-inflammatory therapies and medications that improve endothelial function. Treatment with statins and other lipid-lowering agents can also be beneficial in preventing atherosclerotic plaque formation and reducing chronic inflammation [12,24]. For instance, studies suggest that treatments targeting systemic inflammation and endothelial dysfunction may be crucial in managing patients with a high risk of myocardial infarction and stroke [18,19].

#### 5.3.2. Therapies for Neurological Diseases

For neurological diseases such as Alzheimer’s and Parkinson’s, eradicating *H. pylori* may reduce neuroinflammation and protect neuronal function. In multiple sclerosis, modulating the immune response by eradicating *H. pylori* and using immunomodulatory therapies can help reduce autoimmune attacks on myelin [16,123,141,145]. For example, in Parkinson’s disease, improving motor symptoms by eradicating *H. pylori* can lead to a better quality of life for patients [152]. Additionally, reducing systemic inflammation may have beneficial effects on the progression of Alzheimer’s disease and help maintain cognitive function [16].

In conclusion, *H. pylori* infection is associated with an increased risk of developing cardiovascular and neurological diseases through complex mechanisms involving chronic inflammation, endothelial dysfunction, and autoimmune responses. Eradicating the infection can benefit from reducing the risk and ameliorating the symptoms of these conditions. Continued research is essential to fully elucidate the role of *H. pylori* in these diseases and to develop effective prevention and treatment strategies. Implementing targeted treatments, such as using antibiotics for infection eradication and anti-inflammatory therapies, can positively influence therapeutic approaches and improve clinical outcomes for affected patients.

## 6. Conclusions

This review emphasizes the critical role of *H. pylori* infection beyond gastrointestinal conditions, highlighting its contributions to the development of cardiovascular and neurological diseases. Understanding the complex pathophysiological mechanisms by which *H. pylori* influences these diseases provides new opportunities for therapeutic interventions and preventive measures.

*H. pylori* infection is associated with an increased risk of cardiovascular diseases such as atherosclerosis, myocardial infarction, and stroke, primarily through mechanisms involving chronic inflammation and endothelial dysfunction. In neurological diseases, *H. pylori* may contribute to conditions like Alzheimer’s, Parkinson’s, and multiple sclerosis via systemic inflammation, neuroinflammation, and autoimmune responses. Eradicating *H. pylori* can reduce systemic inflammation and improve endothelial function, potentially lowering the risk and severity of these diseases.

Integrating *H. pylori* screening and eradication into cardiovascular disease management protocols could significantly reduce disease burden. Future research should focus on large-scale clinical trials to validate the effectiveness of such integrated approaches. Further studies are essential to explore the exact mechanisms by which *H. pylori* contributes to neurological diseases. Longitudinal studies could help determine the long-term benefits of *H. pylori* eradication on cognitive function and disease progression. Implementing health policies that promote the early diagnosis and treatment of *H. pylori* infection could have a substantial impact on public health. These policies should emphasize routine screening, especially in populations at high risk for cardiovascular and neurological diseases. Developing personalized treatment plans that consider *H. pylori* infection status could enhance therapeutic outcomes. Research should aim to identify biomarkers that predict response to *H. pylori* eradication in patients with cardiovascular and neurological conditions.

Recognizing and managing *H. pylori* infection represents a significant opportunity for improving patient health outcomes. An integrated clinical approach that includes the evaluation and treatment of *H. pylori* infection can enhance prevention and treatment strategies for cardiovascular and neurological diseases, ultimately improving patient care and quality of life.

## Data Availability

No new data were created or analyzed in this study.
